# Antibacterial and Anti-Inflammatory Activities of *Thymus vulgaris* Essential Oil Nanoemulsion on Acne Vulgaris

**DOI:** 10.3390/microorganisms10091874

**Published:** 2022-09-19

**Authors:** Farah M. Abdelhamed, Nourtan F. Abdeltawab, Marwa T. ElRakaiby, Rehab N. Shamma, Nayera A. Moneib

**Affiliations:** 1 Department of Microbiology and Immunology, Faculty of Pharmacy, Cairo University, Cairo 11562, Egypt; 2 Department of Pharmaceutics and Industrial Pharmacy, Faculty of Pharmacy, Cairo University, Cairo 11562, Egypt

**Keywords:** *Cutibacterium acnes*, *Staphylococcus epidermidis*, inflammation, antibiotic resistance, acne vulgaris, natural antimicrobials, nanoparticles, biofilms, mode of action, plant extracts

## Abstract

Antibiotics are frequently used in acne treatment and their prolonged use has led to an emergence of resistance. This study aimed to investigate the use of natural antimicrobials as an alternative therapy. The antimicrobial and anti-inflammatory activities of five commonly used essential oils (EOs) (tea tree, clove, thyme, mentha and basil EOs), and their possible mechanisms of action against *Cutibacterium acnes* and *Staphylococcus epidermidis*, were explored. The effect of the most potent EO on membrane permeability was elucidated and its anti-inflammatory action, when formulated as nanoemulsion, was tested in an in vivo acne model. The in vitro studies showed that thyme EO had the most potent antimicrobial and antibiofilm activity, with phenolics and terpenoids as main antimicrobial constituents of EO. Thyme EO affected cell membrane permeability of both bacterial species, evident by the detection of the leakage of intracellular ions and membrane integrity by the leakage of nucleic acids. Morphological alteration in bacterial cells was confirmed by transmission electron microscopy. Thyme EO nanoemulsion led to the suppression of an inflammatory response in acne animal models along with a bacterial load decrease and positive histopathological changes. Collectively, thyme EO nanoemulsion showed potent antimicrobial and anti-inflammatory effects compared to the reference antibiotics, suggesting its effectiveness as a natural alternative in acne treatment.

## 1. Introduction

Acne vulgaris is one of the most common chronic inflammatory skin disorders. Almost 85% of individuals suffer from acne during their lifetime [1]. Acne causes significant morbidity and affects patients both physically and psychologically [2]. Acne is characterized by forming inflammatory and non-inflammatory lesions mainly on the face, neck, arms, upper trunk and back [3]. The pathogenesis of acne vulgaris is multifactorial, with contributing factors including a hormonally derived overproduction of sebum (seborrhea), follicular hyperkeratosis, changes in skin microbiota and immunological inflammatory responses [4]. These factors affect each other where seborrhea and increased keratinization of the follicular epithelium produce a favorable environment for the overgrowth of *Cutibacterium acnes* (previously known as *Propionibacterium acnes*) over *Staphylococcus epidermidis* and *Staphylococcus aureus.* Metabolites of these microorganisms induce follicular and perifollicular inflammation, especially due to the release of chemotactic substances [5].

Most anti-acne treatments include the use of antibiotics such as erythromycin and clindamycin [6]. However, these antibiotics can have adverse side effects on the skin’s normal microbiota and hold the potential to cause the emergence of bacteria resistant to these antibiotics. To overcome the emergence of the resistance to conventional antibiotics, alternative natural antimicrobial agents have been tested for their potential effectiveness against acne. Essential oils (EOs) are one of the most important natural products derived from plants for their various biological properties and medicinal uses. EOs have been used around the world for centuries for various purposes. Ancient Egyptians have used aromatic oils as early as 4500 BC in cosmetics and ointments. EOs and their components are active against a variety of targets, particularly the membrane and cytoplasm, and in some cases, they completely change the morphology of the cells [7]. These oils are present as mixtures of various monoterpenes, alcohols, phenols, aldehydes and sulphur-containing compounds. Diverse mechanisms have been studied to elucidate the activity of an EO on bacterial cells. These mechanisms include degradation of the cell wall, damage to the cytoplasmic membrane, coagulation of the cytoplasm and leakage of cell contents due to increased permeability [8]. The hydrophobicity that is typical of EOs is responsible for the disruption of bacterial structures, leading to increased permeability due to an inability to separate the EOs from the bacterial cell membrane [9]. It is well established that *C. acnes* plays a pivotal role in the development of inflammatory skin diseases. Several studies have shown that infection with *C. acnes* involves an interaction with Toll-like receptors TLR-2 and TLR-4 on keratinocyte [10]. Activation of these pattern recognition receptors induces the release of inflammatory cytokines and chemokines, including tumor necrosis factor alpha (TNF-α) and interleukin (IL)-8, which mediate inflammatory responses in both keratinocytes and monocytes [11,12]. Both TNF-α and IL-8 reportedly exacerbate skin inflammation. Therefore, anti-acne treatment should have anti-inflammatory effects besides antimicrobial properties.

This study aimed to test the antimicrobial properties of five EOs commonly used in the Mediterranean region and their potency in treating acne vulgaris. Another goal of the current study was to investigate the possible mode of action of the most effective EO by elucidating its effect on the bacteria cell membrane and intracellular components. The study also aimed to develop a pharmaceutical formulation of the EO with the highest antimicrobial effect. Based on the in vitro antibacterial results of tested EOs against acne-causing bacteria, thyme EO was formulated as a nanoemulsion formula. Healing and anti-inflammatory properties of the developed nanoemulsion of thyme EO were tested in an animal model as a potential new formulation for acne treatment. To the best of our knowledge, this is the first study to explore the mechanism of action of thyme EO in healing acne, in addition to reporting the effective use of thyme EO as a nanoemulsion as a possible alternative anti-acne therapeutic agent.

## 2. Materials and Methods

### 2.1. Bacterial Strains and Their Maintenance

*C. acnes* standard strain ATCC 6919 was obtained from the American Type Culture Collection (ATCC) (Manassas, VA, USA), while *S. epidermidis* DSM 28319 (equivalent to ATCC 35984) was obtained from the German Collection of Microorganisms (Braunschweig, Germany). *C*. *acnes* was cultured anaerobically on RCM agar (Oxoid Limited, Basingstoke, United Kingdom (UK) and incubated for 48 h at 37 °C under ~5% CO_2_ using an anaerobic jar and anaerobic atmosphere generation bags (Sigma–Aldrich, St. Louis, MO, USA). Isolated colonies of *C. acnes* were sub-cultured in an RCM broth for 48 h at 37 °C under anaerobic conditions. For *S. epidermidis*, the bacteria were cultured aerobically on brain heart infusion (BHI) agar (LAB M limited, Lancashire, UK) and incubated at 37 °C for 18 h. Isolated colonies of *S. epidermidis* were sub-cultured in BHI broth and incubated at 37 °C for 18 h aerobically. Glycerol stock of each bacterial strain were prepared using their appropriate media supplemented with 25% glycerol and stored in a −70 °C freezer (Thermo Fisher Scientific, Waltham, MA, USA).

### 2.2. Essential Oils (EOs)

The research-grade EOs used in this study are listed as Table 1 and classified according to the plant family names. The pharmaceutical-grade EOs were purchased from Haraz herbal store (Cairo, Egypt). The composition of the most effective EOs was confirmed by gas chromatography–mass spectroscopy (GC-MS) analysis at the department of pharmacognosy, faculty of Pharmacy, Ain Shams University (Cairo, Egypt), as detailed in Section 2.6.

### 2.3. Animals

A total of 68 adult male BALB/c mice (25–35 g of weight) were used in the current study; mice were purchased from Theodor Bilharz Research Institute (Giza, Egypt). Mice were left to adapt to the study environment for at least two weeks before the start of each experiment. Mice were housed in a controlled environment at 25 ± 2 °C, 55 ± 5% humidity, and 12 h light/dark cycle. Animals were provided with a standard laboratory diet and water ad libitum. The research procedures were conducted in compliance with the principles and recommendations of the Guide for the Care and Use of Laboratory Animals Association, A.V.M (Institute of Laboratory Animal Resources (US) 1986). All the animal experiments were approved by the Research Ethics Committee (REC) of the Faculty of Pharmacy, Cairo University with the protocol identification code MIC2.3.1.

### 2.4. Screening of Antimicrobial Activity of the Selected EOs by Disc-Diffusion Method

As a preliminary step, antibacterial activities of all five EOs were determined using the agar disc-diffusion method according to Kirby–Bauer protocol (CLSI, 2006). EOs were diluted in sterile dimethyl sulphoxide (DMSO) analytical reagent (AR) grade, Loba Chemie (Mumbai, India), and stock solutions of each oil were prepared at a concentration range of 1.56–50%. DMSO and the prepared stock solutions were both sterilized by filtration through 0.2 µm syringe filters (Sigma-Aldrich, St. Louis, MO, USA). Bacterial suspensions of *C. acnes* and *S. epidermidis* were prepared, and optical density was adjusted to 0.5 at OD _600_ nm. Bacterial suspensions were spread on RCM and BHI agar plates for testing EOs against *C. acnes* and *S. epidermidis*, respectively. Sterile filter-paper discs (6 mm in diameter) were loaded with 10 µL of DMSO or EOs at different concentrations (1.56% to 50%) and placed on the surface of the agar, then incubated under appropriate conditions detailed for each bacterial strain, as mentioned in Section 2.1. Standard antibiotics, clindamycin (2 µg/disc) and erythromycin (15 µg/disc), were used as positive controls, while DMSO was used as a negative control. The antibacterial activity of each EO at each concentration was evaluated by measuring the diameter of the zone of inhibition expressed in millimeters (mm). Each assay was performed in triplicate and the whole experiment was repeated at least three times.

### 2.5. Determination of MIC and MBC of the Screened EOs against C. acnes and S. epidermidis

Minimum inhibitory concentration (MIC) and minimum bactericidal concentration (MBC) of EOs were determined using the broth microdilution method in 96-well U-shaped bottom microtiter plates according to the Clinical Laboratory Standards Institute (CLSI)—2011. The EOs were dissolved in sterilized DMSO at a final concentration of 50% (*v*/*v*), then two-fold serial dilutions were performed from 25% to 1.03% (*v*/*v*) for each EO. Bacterial suspensions of *C. acnes* and *S. epidermidis* were prepared in RCM and BHI broth, respectively. The OD _600 nm_ was adjusted to 0.5, then 25 µL of each bacterial suspension, 25 µL of sterile RCM/BHI broth and 50 µL of each concentration of each EO were mixed and incubated at 37 °C for 48 h for *C. acnes* under anaerobic conditions and 24 h for *S. epidermidis* aerobically.

Changes in the bacterial density were measured using a microplate reader at 620 nm. The MIC of the EO was determined as the lowest concentration of EO, at which no detectable bacterial density was observed at OD _600nm_ [13]. Sterile broth and DMSO were used as negative controls. For MBC determination, 10 µL of the mixtures of each bacterial suspension and EOs at different concentrations were inoculated on RCM and BHI agar for *C. acnes* and *S. epidermidis*, respectively. MBC was determined as the lowest concentration of the EO, at which the incubated bacterial strain showed no detectable colonies on its respective agar medium plates. The assays were performed in triplicate and repeated as three independent experiments.

### 2.6. Determination of the Chemical Composition of the Most Effective EOs by Gas Chromatography–Mass Spectroscopy

The chemical components of the most effective EOs were analyzed at the Faculty of Pharmacy Ain Shams University, using GC-MS on a Shimadzu GCMS-QP2010 (Koyoto, Japan) equipped with Rtx-5MS fused bonded column (30 m × 0.25 mm i.d. × 0.25 µm film thickness) (Restek, CA, USA) equipped with a split–splitless injector. The following operating conditions were used where the initial column temperature was kept at 45 °C for 2 min and programmed to 300 °C at a rate of 5 °C/min, and kept constant at 300 °C for 5 min. The injector temperature was 250 °C. The helium carrier gas flow rate was 1.41 mL/min. All the mass spectra were recorded applying the following conditions: filament emission current was 60 mA; ionization voltage at 70 eV; ion source at 200 °C. Diluted samples (1% *v*/*v*) were injected with split mode (split ratio 1: 15). The resultant peaks were identified using AMDIS software by comparing their retention indices (RI), retention times and mass spectra to the authentic samples on the NIST, Wiley library database (>90% match).

### 2.7. Determination of Minimum Biofilm Inhibitory Concentration

The minimum biofilm inhibitory concentration (MBIC) for EOs was determined against *S. epidermidis* using the microtiter plate method [14]. In each well, 100 µL of *S. epidermidis* in BHI–1% glucose (*w*/*v*) with OD _600 nm_ adjusted to 0.5 was mixed with 100 µL of EO at different concentrations 25—0.78% (*v*/*v*) then incubated at 37 °C for 24 h. Following incubation, the contents of the wells were removed and gently rinsed twice with 250 µL of phosphate-buffered saline (PBS). The plate was left to air-dry overnight. The formed biofilm was then stained with 0.5% (*w*/*v*) crystal violet for 30 min at room temperature. The excess crystal violet was washed with 300 µL distilled water per well. The washing step was repeated three times then the plate was air-dried overnight. To measure the stained biofilm, crystal violet was solubilized using ethanol 95% (*v*/*v*) and the color intensity was measured at 595 nm using a microplate reader. Sterile BHI broth was used as a negative control and *S. epidermidis* cell culture without EOs was used as a positive control. MBIC was determined as the concentration of EO, at which the OD _595 nm_ was equal to that of the negative control [15]. Experiments for each EO concentration were performed in triplicate and the assay was repeated three independent times.

### 2.8. Determination of the Minimum Biofilm Eradication Concentration

The minimum biofilm eradication concentration (MBEC), the lowest concentration of EO necessary to completely eradicate preformed biofilm for EOs, was determined against *S. epidermidis* using the microtiter plate method [14]. In each well, 200 µL of *S. epidermidis* suspension in BHI–1% glucose (*w*/*v*) with OD _600 nm_ adjusted to 0.5 were incubated for 24 h at 37 °C to allow biofilm formation. Following incubation, the bacterial suspension was discarded, and the wells were rinsed by flooding with sterile PBS twice then left to air-dry overnight. A total of 200 µL of each EO concentration was added to the wells and incubated at 37 °C for 24 h. After incubation, the contents of the wells were removed and gently rinsed twice with 250 µL of PBS. The plate was left to air-dry overnight and the remaining biofilm was stained with 0.5% (*w*/*v*) crystal violet for 30 min at room temperature. The excess crystal violet was washed with 300 µL of distilled water per well. The washing step was repeated three times then the plate was air-dried overnight. To determine the MBEC, crystal violet was solubilized using ethanol 95% (*v*/*v*) and the color intensity was measured at 595 nm using a microplate reader. The controls were the untreated biofilm and the sterile BHI-1% glucose broth (*w*/*v*).

### 2.9. Determination of Time–Kill Kinetics of Selected EO

The time–kill kinetics of thyme EO against *C. acnes* and *S. epidermidis* were performed according to a previously established protocol [16,17]. Bacterial suspensions at an initial inoculum of 10^8^ CFU/mL were used. The time–kill kinetics of the selected EO were assayed at concentrations of 0.053 mg/mL, 0.106 mg/mL, and 0.212 mg/mL, equivalent to 1, 2 and 4 MIC. Different concentrations of selected EO were incubated with the bacterial cultures and killing capacity at 0, 1, 2, 4, 8 and 12 h, and were assessed using broth micro-dilution method. At each time point, 10 µL of the assay solution was withdrawn to make ten-fold serial dilutions and subjected to viable colony counts on BHI and RCM agar plates for the respective bacteria. Plates were incubated at 37 °C for 24 h under aerobic conditions and 48 h under anaerobic conditions for each respective bacterial strain. DMSO, BHI broth and RCM broth were used as negative controls. Each concentration of EO was assayed as triplicate and the entire assay was repeated two independent times.

### 2.10. Assessment of Possible Mechanisms of Action of Selected EO against Acne Associated Microbes

The most effective selected EO was assayed for its possible anti-acne mechanism of action using multiple assays, including induced morphological alterations in the tested bacteria, loss of membrane integrity and leakage of intracellular components.

#### 2.10.1. Observation of Morphological Alteration of *C. acnes* and *S. epidermidis* Treated with Selected EO

##### Visualization of the Effect of the Selected EO on Biofilm by Scanning Electron Microscopy

*C. acnes* and *S. epidermidis* suspensions were incubated overnight in appropriate broth media supplemented with 1% glucose at 37 °C. Following incubation, bacterial density was adjusted to 0.5 at OD _600 nm_. A clear sterile cover slide was then added to each well of a six-well plate. In one set of wells, 250 µL of the selected EO at its MBIC concentration was added to an equal volume of *C. acnes* or *S. epidermidis* and incubated at 37 °C for 48 h and 24 h, respectively. In another set of wells, 250 µL of DMSO was added to an equal volume of *C. acnes* or *S. epidermidis* and served as the negative control. Following incubation, cover slides were gently washed with saline and prepared for SEM photography using JEOL model JSM-5200F electron microscope 25 kV, with a resolution of 5.5 nm and magnification power of 15× to 200,000×, JEOL Ltd. (Tokyo, Japan) at the Faculty of Agriculture, Cairo University, Cairo, Egypt.

##### Visualization of the Effect of the Selected EO on Bacterial Cells by Transmission Electron Microscopy

Cellular morphological alterations of bacteria treated with the selected EO was observed by transmission electron microscopy (TEM). Bacterial suspensions of *C. acnes* and *S. epidermidis* were prepared, their OD _600 nm_ was adjusted to 1 then the MBC of the selected EO was added to each suspension and incubated for 6 h. The bacterial suspensions were then centrifuged and washed twice using a PBS and treated with 2.5% glutaraldehyde overnight. The samples were prepared according to a previously published protocol [18] and observed by TEM photography using JEOL model JEM-1400Flash electron microscope 120 kV, with a resolution of 0.2 nm and magnification power of 10× to 1,200,000×, JEOL Ltd. (Tokyo, Japan) at the Faculty of Agriculture, Cairo University, Cairo, Egypt.

#### 2.10.2. The Effect of the Selected EO on Bacterial Membrane Integrity

##### The Effect of the Selected EO on Potassium Ions Permeability

Bacterial suspensions of *C. acnes* and *S. epidermidis* were prepared according to a previously published protocol [19]. Isolated colonies (5–7 colonies) of *C. acnes* and *S. epidermidis* were inoculated in RCM and BHI broth, respectively, then incubated at 37 °C for 48 h and 24 h, respectively. Following incubation, bacterial cells were centrifugated at 10,000 rpm for 10 min and washed twice with PBS and resuspended in saline. The optical density was adjusted at 600 nm to 1. The bacterial cells were then treated with the MIC of the selected EO or DMSO (negative control) for 6 h and centrifuged at 10,000 rpm for 10 min. After centrifugation, the amounts of potassium (K^+^) were measured in the supernatant using the ICP spectrometry technique [20] at the Faculty of Agriculture, Ain Shams University, Cairo, Egypt.

##### The Effect of the Selected EO on 260 nm Absorbing Material (Nucleic Acids)

The loss of nucleic acids through increased membrane permeability was assessed according to a previously published protocol [21] with some modifications. Bacterial suspensions of *C. acnes* and *S. epidermidis* were prepared. Isolated colonies (5–7 colonies) of *C. acnes* and *S. epidermidis* in RCM and BHI broth were incubated at 37 °C for 48 h and 24 h, respectively. After incubation, the bacterial suspensions were centrifuged at 10,000 rpm for 5 min, washed twice with PBS then resuspended in saline to adjust the optical density at 600 to 1. Resuspended bacteria in saline were treated with either DMSO or clindamycin (Dalacin C 600 mg/4 mL ampule, Pfizer) as negative controls, whereas vancomycin (Vancomycin 500 mg/10 mL ampoule, Mylan) was used as a positive control. The selected EO was tested at a final concentration equivalent to its MIC. Samples from bacterial cultures under each treatment were withdrawn after 30, 60 and 120 min, and were filtered through a 0.2-μm filter. The OD _260 nm_ of each test filtrate was measured using UV-Visible Spectro nanophotometer (IMPLEN GmbH, Munich, Germany).

##### The Effect of the Selected EO on Leakage of Intracellular Ions

The leakage of ions was assessed according to a previously published protocol [19] with minor modifications. Bacterial suspensions of *C. acnes* and *S. epidermidis* were prepared. Isolated colonies (5–7 colonies) of *C. acnes* and *S. epidermidis* were inoculated in RCM and BHI broth then incubated at 37 °C for 48 h and 24 h, respectively. Following incubation, cells were centrifugated at 10,000 rpm for 10 min and washed twice with PBS; the optical density was adjusted at 600 nm to 1. The bacterial cells were then treated with the MBC of the selected EO or DMSO (negative control) for 6 h and centrifuged. After centrifugation, the amounts of phosphate (PO^4−^) and sulphur (S^2−^) ions were measured using the ICP spectrometry technique [19] at the Faculty of Agriculture, Ain Shams University, Cairo, Egypt.

### 2.11. Development and Characterization of the Selected EO Nanoemulsion

#### 2.11.1. Development of the Selected EO Nanoemulsion

Nanoemulsion was prepared by a low-energy method [22] using 41.85% (*w*/*w*) of water, 2 MIC of the selected EO, tween 80 (27.5% *v*/*v*), and PEG 400 (27.5% *v*/*v*) as the surfactant and co-surfactant, respectively. The EO, tween 80, and PEG 400 were stirred at 800 rpm using a magnetic stirrer (Fisatom, Brazil) for 30 min. Water was then added dropwise, and the mixture was stirred at 800 rpm for 60 min. In the current study, we formulated thyme EO, clindamycin 1% (positive control) and blank formula (negative control) into nanoemulsions. The nanoemulsions were stored at room temperature (20 ± 2 °C) and evaluated after 1, 7, 21 and 30 days of preparation.

#### 2.11.2. Characterization of EO Nanoemulsion

##### Determination of the Particle Size and Polydispersity Index

The size and size distribution of thyme oil nanoemulsion were determined by the dynamic light scattering method [23] using a Malvern Mastersizer (DLS, Zetasizer Nano ZS, Malvern instruments, Malvern, UK). The polydispersity index (PDI) was measured using Malvern instruments to assess the particle size distribution. Three samples were used for size determination and the average values ± SD were calculated.

##### Nanoemulsion Morphology Using TEM

The morphology of the prepared thyme oil nanoemulsion was characterized using ouldn’tTEM at an acceleration voltage of 80 KeV (model JEM-1230, JEOL, Tokyo, Japan), where one drop of the diluted microemulsion was dried on the surface of a carbon-coated copper grid then stained with 2% phosphotungstic acid and allowed to dry at room temperature for 10 min for TEM inspection [24].

### 2.12. In Vivo Acne Animal Model for Assessment of EO Nanoemulsion Efficacy

#### 2.12.1. Assessment of the Irritability of the EO Nanoemulsion

A preliminary experiment was performed to examine the possible irritability of the prepared nanoemulsion. In this preliminary experiment, 10 µL of the prepared nanoemulsion was applied epicutaneously to the ears of a group of healthy uninfected mice (n = 5 BALB/c mice). Mice were observed over 5 days for any signs of inflammation [25].

#### 2.12.2. Experimental Design

For the assessment of the selected EO nanoemulsion efficacy in vivo, 63 BALB/c male mice were used in three separate experiments (n = 21). In each experiment, mice were divided into three groups (n = 7 mice/group/experiment). Acne infection and inflammation were induced by injecting the right ear with 20 µL of 10^10^ CFU/mL *C. acnes* according to a previously established protocol [26] using a Hamilton syringe 50 µL model 705 RN (Reno, NV, USA). The microcomedone formation was observed 24–48 h after injection. Daily changes in the ear thickness were recorded using an electronic digital micrometer caliper (0–25 mm/0.001 mm). The appearance of microcomedones and the increase in mice ear thickness by ≥10% were considered as indicators of acne induction [26]. For infected mice ears, 20 µL of the prepared nanoemulsion (test group) was applied epicutaneously, or 1% clindamycin (positive control), or blank nanoemulsion formula (negative control) for 3–5 days. Daily changes in mice ear thickness and the general health of the mice were recorded. At the end of the experiment, the mice were euthanized, and mice ears were excised for viable bacterial counts, an inflammatory markers assessment and histopathological assay (Olympus BX50 microscope, Tokyo, Japan) at a magnification power of 400×. Digital photographs of mice ears were taken.

#### 2.12.3. Assessment of the Anti-Inflammatory Activity of the Selected EO Nanoemulsion

The percent of post-treatment epicutaneous inflammation was calculated for all the animal groups using the following formula:

(1) $$\%\mathrm{inflammation}\;=\;\frac{{\mathrm{ear}\;\mathrm{thickness}}_{\mathrm{infected}\;\mathrm{ear}}\;-\;{\mathrm{ear}\;\mathrm{thickness}}_{\mathrm{uninfected}\;\mathrm{ear}}}{{\mathrm{ear}\;\mathrm{thickness}}_{\mathrm{infected}\;\mathrm{ear}}}$$

The percentage inhibition of inflammation for all the animal groups was calculated as described previously [27] using the following formula:

(2) $$\%\mathrm{inflammation}\;\mathrm{inhibition}\;=\;\frac{{\%\mathrm{inhibition}}_{\mathrm{control}}\;-\;{\%\mathrm{inhibition}}_{\mathrm{treatment}}}{{\%\mathrm{inhibition}}_{\mathrm{control}}}$$

#### 2.12.4. Assessment of the Anti-Inflammatory Activity of the Selected EO Nanoemulsion on Inflammatory Mediators

The NF-κB was measured in mice ear tissue homogenate using the Mouse Nuclear Factor KB (NFKB) ELISA Kit (MyBio Source, San Diego, CA, USA). The samples were prepared according to the kit manual and NF-κB concentration was determined and compared among different treatment groups.

#### 2.12.5. Histopathological Examination of Ears after Treatment with EO Nanoemulsion

For the histopathological examination, whole excised mice ears in the 10% formalin solution were preserved before preparing the histological sections using the paraffin method technique [28]. All sections in ascending grades of ethanol were dehydrated, cleared in xylene then embedded in paraffin wax. Transverse sections (4–5 µm, thickness) were mounted on glass slides and stained with hematoxylin and eosin (H&E) stains. All sections were examined for the evaluation of inflammatory responses.

#### 2.12.6. Assessment of the Antimicrobial Activity of EO Nanoemulsion

For an in vivo assessment of the antimicrobial activity of the prepared nanoemulsion, excised mice ears were homogenized in saline. The homogenates were then diluted using different dilutions of bacterial suspension, and 10 µL was cultured on RCM agar plates. *C. acnes* colonies were counted after 48 h of anaerobic incubation at 37 °C. Bacterial counts were expressed as CFU/mL.

### 2.13. Statistical Analysis

Data were plotted and analyzed using GraphPad Prism 9, GraphPad Software Inc. (GraphPad Prism version 9.0.0 (86) for OS X (GraphPad Software, San Diego, CA, USA, www.graphpad.com). Data were presented as mean ± standard deviation (SD) of at least three independent biological experiments. A one-way ANOVA test with Tukey’s multiple comparison was used for the analysis of the results of agar disc-diffusion, reduction in NF-KB among different treatment groups and the antimicrobial activity between EO- and clindamycin-treated mice in the in vivo experiment. The unpaired t-test was performed to compare the results of the effect of EO on membrane integrity and the leakage of intracellular ions after treatment of bacteria with EO, whereas the two-way ANOVA test was applied to assess the statistical difference between EO and standard antibiotics in the loss of nucleic acid. The Mann–Whitney test was performed to compare the effect of the EO and clindamycin nanoemulsions in lowering the rate of inflammation in the animal model. Significance was considered at *p*-value ≤ 0.05.

## 3. Results

### 3.1. Screening of the Antimicrobial Activity of the Tested EOs by Disc- Diffusion Method

**Figure 1:**
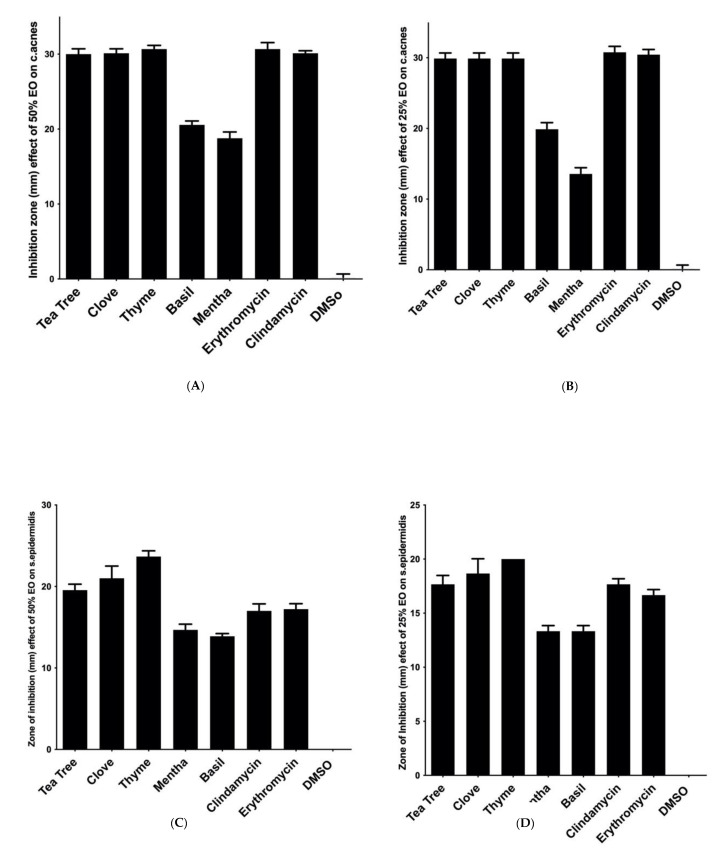
Antibacterial activity of the screened essential oils (EOs) using the agar disc-diffusion method against (**A**) *Cutibacterium acnes* with EOs at a concentration of 50% *v*/*v*; (**B**) *C. acnes* with EOs at a concentration of 25% *v*/*v*; (**C**) *Staphylococcus epidermidis* with EOs at a concentration of 50% *v*/*v*; (**D**) *S. epidermidis* with EOs at a concentration of 25% *v*/*v*. Data are represented as means of inhibition zones (mm) ± standard deviation (SD). Controls used were clindamycin and erythromycin as positive controls, while dimethyl sulphoxide (DMSO) was used as a negative control.

Tea tree, clove and thyme EOs exhibited the largest inhibition zones among the screened EOs using the agar disc-diffusion method, while DMSO showed no zone of inhibition against both bacterial strains. The effect of tea tree, clove and thyme EOs at 25% and 50% (*v*/*v*) against *C. acnes* was not significantly different from the standard antibiotics. However, the three mentioned EOs were significantly more effective than the standard antibiotics at 25% and 50% (*v*/*v*) against *S. epidermidis p*-value ≤ 0.05 (Figure 1).

### 3.2. Determination of the Minimum Inhibition Concentration and the Minimum Bactericidal Concentration of the Screened EOs

The MIC of the selected EOs was determined as the lowest concentration of the oils, resulting in bacterial inhibition. The most potent EOs against *C. acnes* and *S. epidermidis* were tea tree and thyme, showing MIC values against *C. acnes* of 0.053 g/mL and 0.026 g/mL, respectively (Table 2). Tea tree and thyme EOs also showed the lowest MIC values of 0.053 g/mL against *S. epidermidis*. MBC results showed that tea tree and thyme EOs exhibited killing of the bacteria at concentrations equal to their MIC against both bacteria (Table 3). These two oils showed the lowest MBC values against *C. acnes* and *S. epidermidis* compared to other EOs.

### 3.3. Determination of Chemical Composition of the Most Effective EOs by Gas Chromatography–Mass Spectroscopy

The GC-MS analysis of tea tree, clove and thyme EO constitutes showed that the most abundant volatile compound of tea tree was 4-terpinenyl acetate (66.82%) (Supplementary Table S1, Supplementary Figure S1), while eugenol represented the major constituent of clove oil (78.61%) (Supplementary Table S2, Supplementary Figure S2). Thymol constituted 75.46% of thyme oil (Table 4, Supplementary Figure S3).

### 3.4. Determination of the Minimum Biofilm Inhibitory and Eradication Concentration of the Most Potent EOs

The selected EOs were tested for their anti-biofilm activity against *S. epidermidis*. Thyme EO showed the highest biofilm inhibition at half of its MIC of 0.053 g/mL. However, tea tree and clove EOs had anti-biofilm activity against biofilm formed by *S. epidermidis* at double their MIC of 0.107 g/mL and 0.274 g/mL, respectively (Table 5). Moreover, EOs were assayed for their anti-biofilm activity against *S. epidermidis* preformed biofilm. Thyme EO showed the highest biofilm eradication at its MIC of 0.107 g/mL, while clove EO had anti-biofilm activity against the biofilm formed by *S. epidermidis* at double of its MIC of 0.274 g/mL. However, tea tree oil was the least effective EO as it did not affect the preformed biofilm of *S. epidermidis* at a concentration of up to 0.215 mg/mL (4 its MIC) (Table 5).

### 3.5. Determination of Time–Kill Kinetics of Selected EO

**Figure 2:**
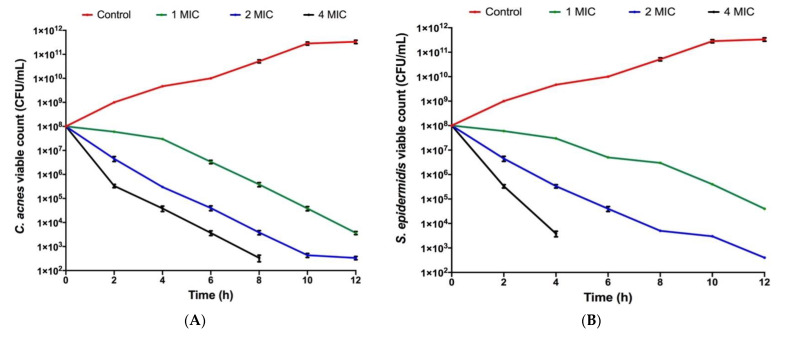
Time–kill assay curves of thyme essential at 1, 2 and 4 MIC against (**A**) *C. acnes,* where it exerted its bactericidal activity after 10 h and (**B**) *S. epidermidis,* where thyme EO exerted its bactericidal effect after 6 h. All data are represented as superimposed dots. The experiment was performed in triplicate and the assay was repeated two independent times.

The antimicrobial testing results proves that thyme EO had the strongest antimicrobial activity and antibiofilm effect compared to the other tested EOs. Hence, thyme EO was selected for further analysis and characterization. Thyme EO had a bactericidal action after 10 h and 6 h of incubation against both *C. acnes* and *S. epidermidis*, respectively. The killing efficacy of thyme EO on *S. epidermidis* was higher than that of *C. acnes* at 6, 8 and 12 h (Figure 2).

### 3.6. Assessment of Possible Mechanisms of Action of Thyme EO on Acne-Associated Microbes

#### 3.6.1. Observation of Morphological Alternations of *C. acnes* and *S. epidermidis* Treated with Thyme EO

##### Visualization of the Effect of the Thyme EO on Bacterial Cells by Transmission Electron Microscopy

**Figure 3:**
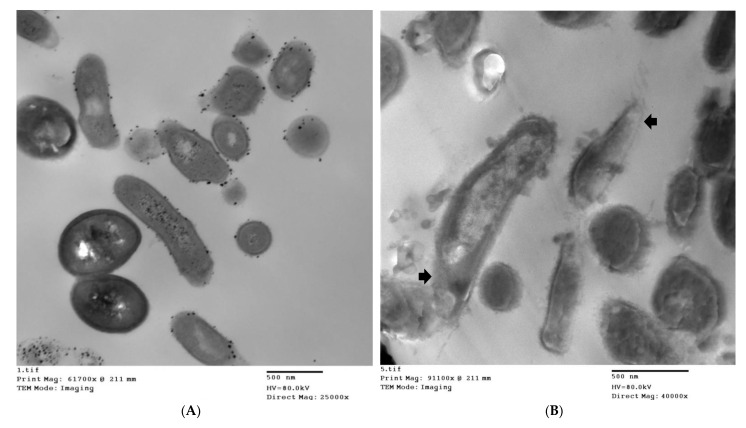

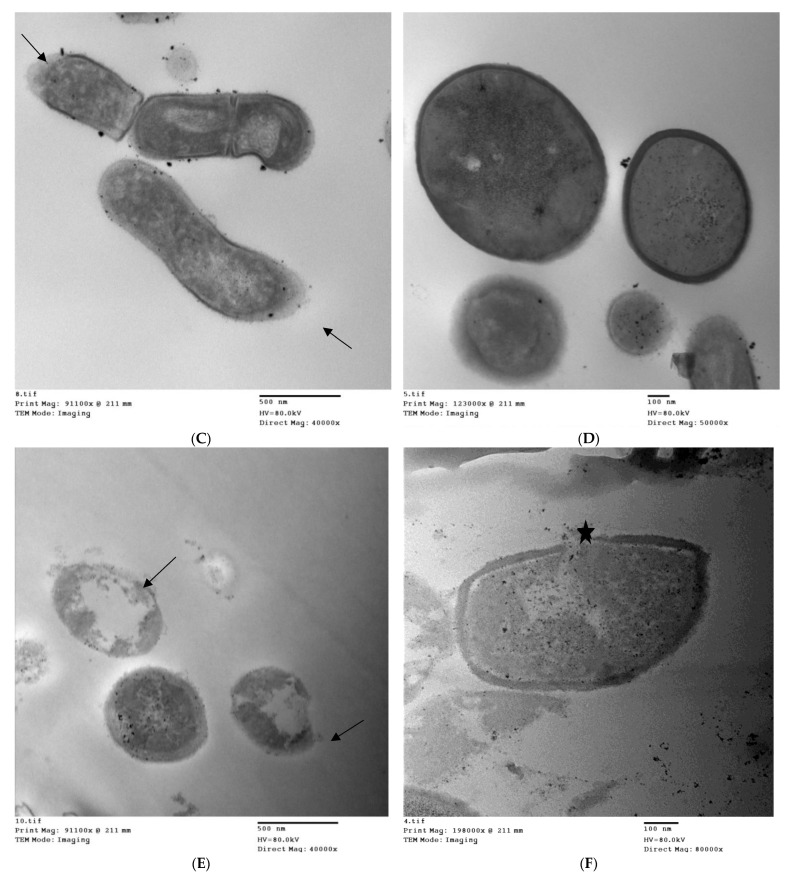
Transmission electron microscope (TEM) photographs of (**A**): *C. acnes* control bacteria with an intact cell wall and intracellular content, (**B**,**C**): *C. acnes* after treatment with MIC of thyme EO, (**D**): *S. epidermidis* control bacteria, (**E**,**F**): *S. epidermidis* after treatment with thyme EO. The arrows show the leakage in the cytoplasm while the thick arrowheads indicate the cellular damage, and the star shows pore formation in the cell membrane. Notice the amorphous vacuolated cells.

The mechanism of action of thyme EO on membrane permeability was shown in TEM photographs of *C. acnes* and *S. epidermidis* (Figure 3). Treatment of bacteria with thyme EO at its MBC led to bacterial cell wall damage and leakage of cytoplasmic content. TEM results suggested that thyme EO interferes with bacterial membrane integrity.

##### Visualization of the Effect of Thyme EO on Biofilm Formation by Scanning Electron Microscopy

The SEM was chosen for analyzing the surface and morphological changes in biofilm cells exposed to thyme EO. Regarding *C. acnes* and *S. epidermidis* biofilm formation, cellular adhesion and aggregation were detected in the untreated biofilm (control) (Supplementary Figure S4A,C); however, for biofilm treated with thyme EO, a lower number of adherent cells were observed (Supplementary Figure S4B,D).

#### 3.6.2. Effect of Thyme EO on Bacterial Membrane Integrity

##### Effect of Thyme EO on Potassium Ion (K^+^) Permeability

**Figure 4:**
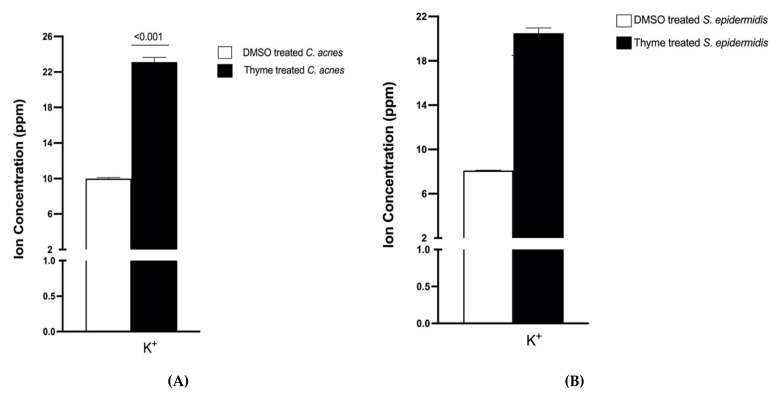
Effect of thyme EO on membrane integrity through the leakage of K+ ion from (**A**) *C. acnes* and (**B**) *S. epidermidis*. Thyme EO at its MBC resulted in a significant release of K+ ion compared to DMSO negative control. The means ± SDs for three replicates are illustrated; an untreated t-test was applied with *p*-value < 0.001.

The effect of thyme EO on the membrane integrity of bacterial cells was assessed through the leakage of potassium ions (K^+^), as shown in Figure 4. Treatment of *C. acnes* and *S. epidermidis* with thyme EO at its MBC for 6 h has led to an observed leakage of K^+^.

##### Effect of Thyme EO on 260 nm Absorbing Material (Nucleic Acids)

**Figure 5:**
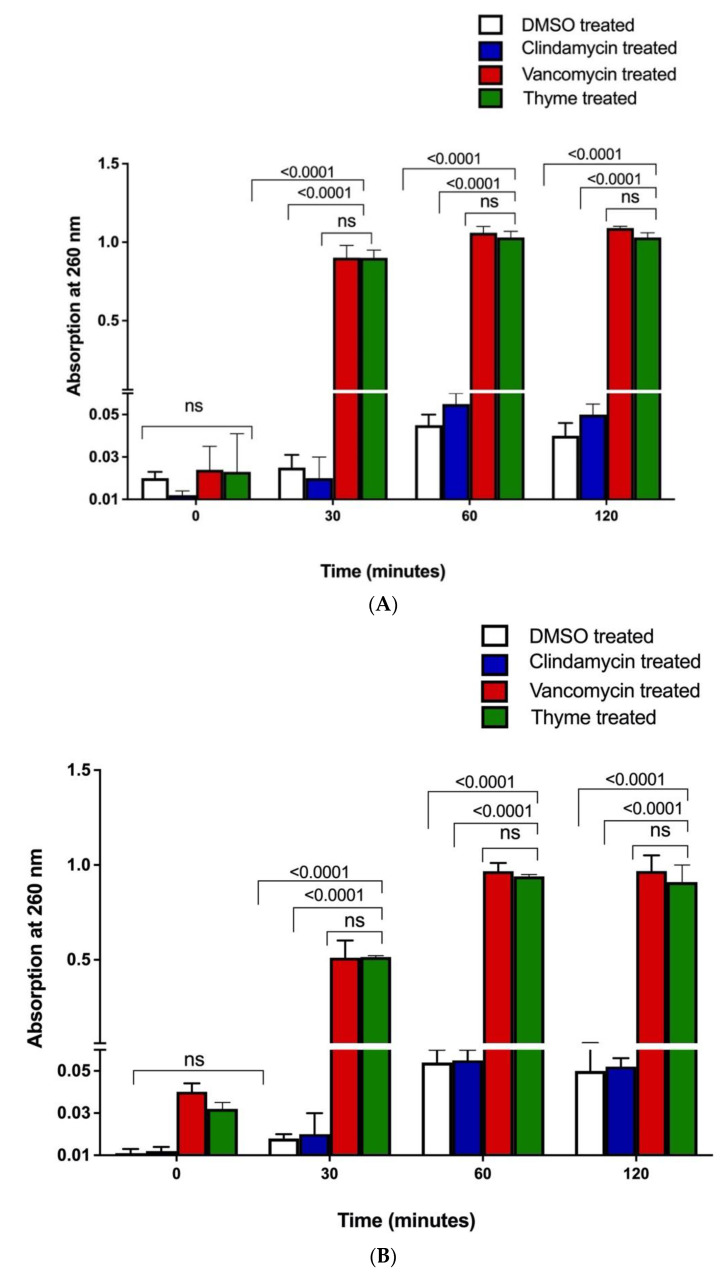
Loss of nucleic acids. The appearance of 260 nm of absorbing material in the filtrates of (**A**) *C. acnes* and (**B**) *S. epidermidis* control suspensions (white bars) and after treatment with clindamycin (silver bars), vancomycin (grey bars) and the MICs of thyme EO (black bars) confirms the effect of thyme EO on bacterial membrane integrity. The means ± SD for at least three replicates are illustrated. A two-way ANOVA test was performed, *p*-value < 0.0001. ns means not statistically significant.

The OD _260nm_ of filtrates from control suspensions were not significantly different after 30, 60 and 120 min. Significant increases in the OD _260nm_ occurred after 30 min of treatment with thyme EO (Figure 5).

#### 3.6.3. Effect of Thyme EO on the Leakage of Intracellular Ions

**Figure 6:**
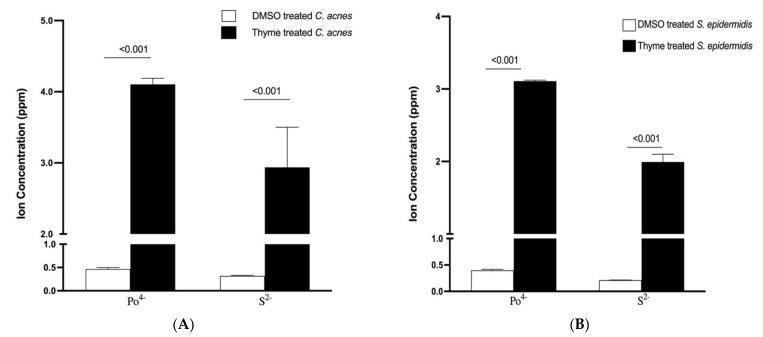
Extracellular concentration of phosphate (PO^4−^) and sulfur ions (S^2−^) in aliquots of *C. acnes* (**A**) and *S. epidermidis* (**B**) after treatment with DMSO (control) and thyme EO at its MBC for 6 h. Data are represented as mean ± SD of three independent experiments. A multiple unpaired *t*-test test was performed, *p*-value < 0.001.

The effect of adding thyme EO at its MIC on the phosphorus (PO^4−)^ and sulfur (S^2−^) leakage in both *C. acnes* and *S. epidermidis* is shown in Figure 6. Compared to the DMSO control, leakage was observed after EO addition. These results suggest that increased membrane permeability is a factor in the mechanism of antimicrobial action.

### 3.7. Development and Characterization of Thyme EO Nanoemulsion

#### 3.7.1. Determination of the Particle Size and Polydispersity Index (PDI)

The z-average diameter recorded for the prepared microemulsion was 77.32 nm with a PDI of 0.38. This small particle size, together with its narrow distribution, provides high microemulsion stability, indicates the presence of droplet microemulsion and explains the clarity and isotropicity of the formulation, as shown in Supplementary Figure S5.

#### 3.7.2. Nanoemulsion Particles Morphology Using TEM

The TEM micrograph of the prepared thyme oil nanoemulsion is illustrated in Supplementary Figure S6. Dark droplets were observed, almost spherical in shape, with the possibility to notice the outermost layer. Dark droplets were observed probably due to the strong interaction between the oil and the phosphotungstic acid used in staining the droplets.

### 3.8. In Vivo Acne Animal Model for Assessment of EO Nanoemulsion Efficacy

**Figure 7:**
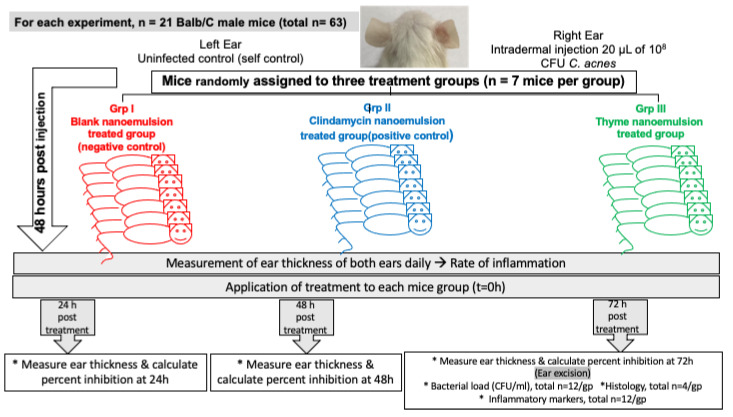
Diagrammatic representation for the in vivo experiment.

Essential oil dose in the formulation was adjusted to 2 MICs (0.052 mg/mL) to avoid possible irritation or hypersensitivity on skin. The anti-acne activity of the formulated nanoemulsion of thyme EO was assessed using an in vivo acne mouse model. BALB/c right ears of mice were injected intradermally with *C. acnes*. After two days, the thyme nanoemulsion, 1% clindamycin or blank formulae was applied epicutaneously on the left ears of mice, and anti-inflammatory and antimicrobial activity against *C. acnes* were assessed (Figure 7).

#### 3.8.1. Assessment of the Irritability of the Thyme Nanoemulsion

To avoid any possible hypersensitivity skin reaction, thyme nanoemulsion was applied to mice ear tissue before initiating the in vivo experiment. Mice were observed over five days for any sign of inflammation. Animals showed no inflammation or irritability when the thyme nanoemulsion formula was applied to healthy uninfected mice ears.

#### 3.8.2. Morphological and Histopathological Assessment of the Healing Activity of the Nanoemulsion

**Figure 8:**
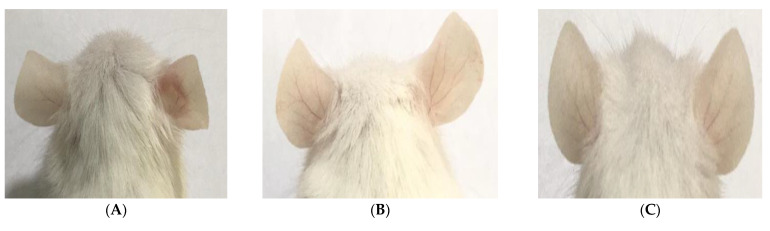

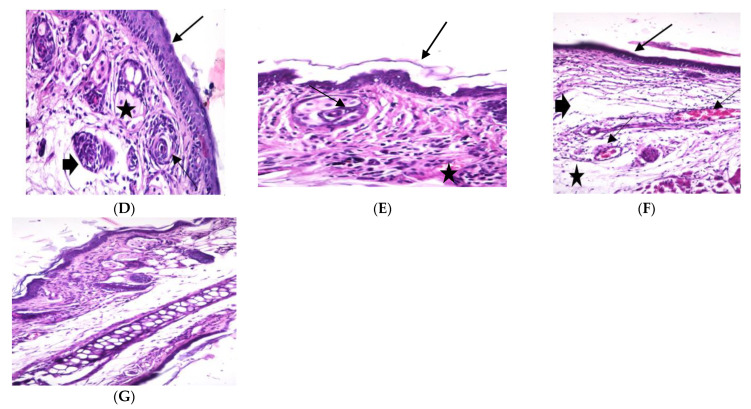
Morphological and histopathological changes in BALB/c mice ear skins at the end of the experiment. (**A**) Appearance of microcomedones and inflammation in the right mouse ear in blank formula-treated mice (negative control); (**B**) absence of inflammatory signs in the right mouse ear treated with thyme formula; (**C**) absence of inflammation in the right mouse ear treated with clindamycin (positive control); (**D**) normal mouse ear tissue with basal layer and epidermal cell maturation was preserved; squamous epithelium (thick arrow), hair follicles (thin arrow), sebaceous glands (*), and subcutaneous tissue (arrow head) were normal, (H&E ×200); (**E**) mouse ear tissue treated with thyme EO nanoemulsion; normal epidermal thickening (thick arrow) with markedly reduced inflammatory cells infiltration in the dermis (*), hair follicles and sebaceous glands were normal (thin arrow), (H&E ×400); (**F**) mouse ear tissue treated with blank formula; epidermal hypoplasia (thick arrow) and dermal thickening with edema (arrow head), note the congested blood vessel (thin arrow), and mononuclear cells infiltrations (*), (H&E ×200) and (**G**) mouse ear tissue treated with 1% clindamycin formula showing skin squamous epithelial cells show proliferous thickening (arrow) and the subcutaneous tissue; thickening with edema (arrow head) and small amount of (inflammatory cells invasion (*), (H&E ×100).

Photographic pictures of BALB/c mice ear skins at the end of the experiment showed gross healing in mice treated with the thyme nanoemulsion. Moreover, the histopathological analysis of mice ears showed that thyme nanoemulsion was a potent anti-acne agent, as mice ears were completely healed. No necrotic dermatitis was detected, and normal dermatitis was observed after the thyme nanoemulsion was applied (Figure 8 and Table 6).

#### 3.8.3. Assessment of Anti-Inflammatory Activity of the Thyme Nanoemulsion

**Figure 9:**
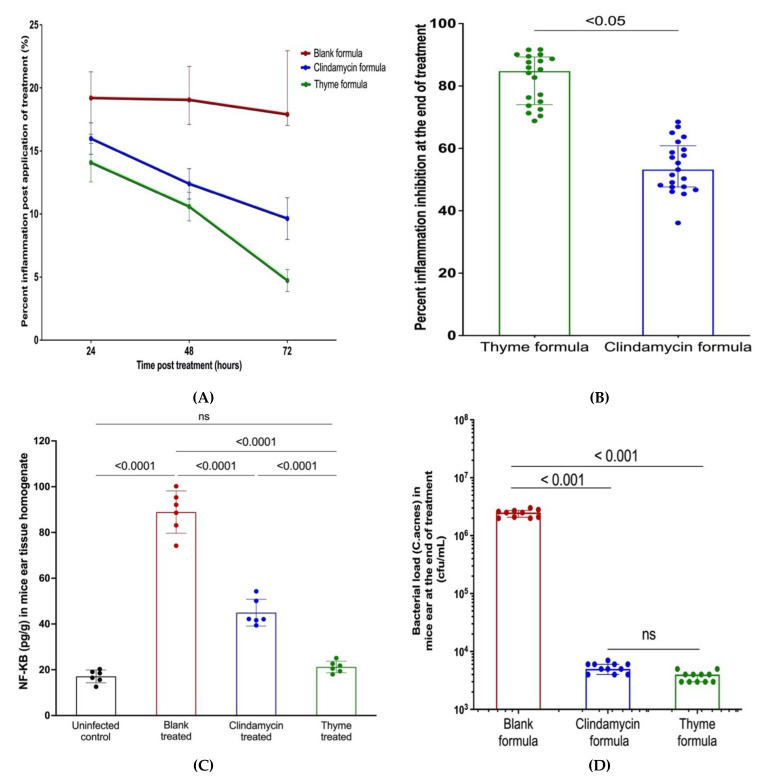
(**A**) Rate of inhibition of inflammation by thyme EO nanoemulsion and controls in acne mouse model showing that the reduction in mice ear thickness post-treatment with thyme EO was superior to clindamycin as a positive control; (**B**) Percent of inflammation inhibition at the end of treatment showing thyme EO nanoemulsion resulting in more than 80% inhibition of inflammation at the end of the treatment period, while clindamycin resulted in a reduction in inflammation by 55%. Mann–Whitney test was performed, *p*-value < 0.05; (**C**) Thyme EO resulted in a 5-fold reduction in NF-κB levels while clindamycin caused only 2.5-fold reduction in the transcription protein. Ordinary one-way ANOVA—Tukey’s multiple comparison test was performed, *p*-value < 0.001; (**D**) In vivo antimicrobial activity of thyme EO nanoemulsion and clindamycin control exerting potent antimicrobial activity against *C. acnes* as the bacterial load was reduced to ~10^3^ cfu/mL in both groups, while the bacterial load in the blank formula-treated mice group (negative control) was kept at 10^5^ cfu/mL. Ordinary one-way ANOVA—Tukey’s multiple comparison test was performed, *p*-value < 0.0001. Data are represented as mean ± SD of three independent experiments.

The percentage of inflammation inhibition was calculated for all animal groups. The rate of reduction in mice ear thickness post-treatment with thyme EO was superior to the clindamycin-positive control (Figure 9). Moreover, inhibition of inflammation at the end of the experiment was significantly higher in mice treated with thyme nanoemulsion compared to the clindamycin-positive control (Figure 9).

#### 3.8.4. Assessment of Anti-Inflammatory Activity of the Thyme Nanoemulsion by Measuring NF-κB Levels

NF-κB concentration in mice ear tissue was determined using the ELISA kit. The result showed no significant difference between the thyme-treated mice group and the healthy uninfected mice group. Moreover, thyme nanoemulsion resulted in a significant 5-fold reduction in NF-κB level, whereas clindamycin nanoemulsion led to only a 2.5-fold reduction in NF-κB. This result confirms the anti-inflammatory effect of the EO (Figure 9).

#### 3.8.5. Assessment of the Antibacterial Activity of the Prepared Nanoemulsion

The in vivo antimicrobial activity of thyme EO nanoemulsion against *C. acnes* was tested. Bacterial load was expressed as Log10 CFU/mL. There was no significant difference between thyme EO and clindamycin nanoemulsions regarding the antimicrobial activity, as both resulted in a reduction in the bacterial count compared to the blank formula (Figure 9).

## 4. Discussion

The microbial loads of *C. acnes* and *S. epidermidis* were found to increase simultaneously in acne vulgaris, which indicates their important role in the development and regulation of acne disease [29,30]. Therefore, *C. acnes* and *S. epidermidis* were chosen to be the targets for the assessment of the anti-acne natural drugs. The mainstay treatment of acne vulgaris involves the use of antibiotics such as erythromycin and clindamycin through their effects on acne-causing microbes [31,32,33]. However, the overuse of antibiotics in acne treatment is associated with the risk of emerging antibiotic resistance. Consequently, to overcome antibiotic resistance and to minimize the high cost of treatment, plants have been studied as an alternative therapy for acne.

In the search for alternative medicines, researchers sought natural products such as EOs, which have been used extensively as pharmaceutical agents for the management of various diseases including acne vulgaris with no reported antimicrobial resistance. EOs are secondary metabolites produced by many aromatic plants that contain a mixture of numerous types of bioactive molecules [34]. EOs have potent antimicrobial properties against a wide spectrum of pathogens including Gram-positive and Gram-negative bacteria. In a previous study, several EOs, including eucalyptus, tea tree, thyme white, lavender, lemon, lemongrass, cinnamon, grapefruit and clove buds were tested against the *Staphylococcus aureus*, Streptococci and *Candida* strain, and the EO of thyme white, lemon, lemongrass and cinnamon oil demonstrated that they were effective against these problematic bacteria [35].

In this study, we investigated the antimicrobial and anti-inflammatory activity of five of the most commonly used EOs belonging to the family Lamiaceae and Myrtacaea, and their mechanisms of action as anti-acne agents. Tea tree EO is currently used commercially in anti-acne OTC products [36] with known anti-acne activity. Three of the selected EOs—thyme, basil and mentha—belong to the Lamiaceae family, known for having members with antimicrobial activity.

Thyme, tea tree and clove EOs inhibited the growth of *C. acnes* and *S. epidermidis* when tested by the disc-diffusion method. These results are in accordance with the study by [33], where *Oregano vulgare* and *Thymus vulgaris* oils showed bacteriostatic effects against Gram-positive and Gram-negative bacterial strains. It was also proven that *Oregano vulgare* and *Thymus vulgaris* oils were more potent than *Ocimum basilicum* oil, because the latter contains estragole, which lacks the antibacterial properties of thymol and carvacrol contained in the former oils [37].

The bactericidal activities of thyme, clove and tea tree EOs were confirmed by the MBC values similar to their corresponding MICs (Table 2 and Table 3) as well as the time–kill kinetics curves (Figure 2). The strongest and fastest bactericidal effect was shown by thyme EO, causing a total elimination of the initial bacterial inoculum after 10 and 6 h of exposure against *C. acnes* and *S. epidermidis*, respectively. In a previous study, the in vitro anti-acne potentials of tea tree, eucalyptus and thyme oils were also validated against *C. acnes* and *S. epidermidis* as antibacterial agents [38].

In the pathogenesis of acne, *S. epidermidis* plays a role in biofilm formation. We, therefore, assessed the anti-biofilm activity of the most potent EOs. Our results show that thyme EO possessed the strongest anti-biofilm activity.

The high potency of thyme EO can be attributed to its lipophilic property and its constituents which target the bacterial membranes [39]. The analysis by GC-MS of thyme EOs showed that thymol was its principal phenolic component (>70%) (Table 4).

**Figure 10:**
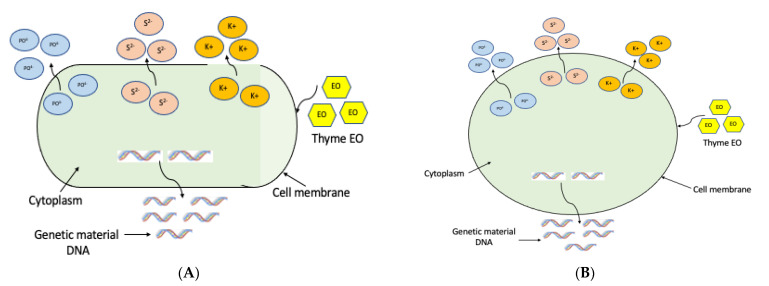
Schematic diagram of mechanism of action of thyme EO against (**A**) *C. acnes* and (**B**) *S. epidermidis*, showing that the drug affects bacterial cell membrane, leading to a leakage of cytoplasmic components, including genetic material and intracellular ions.

Several studies have attempted to elucidate the modes of action of EOs; however, the antibacterial mechanisms are still not clear. Some EO constituents have been shown to penetrate the peptidoglycan layer and act on the cytoplasmic membrane of bacteria. As a result, a leakage of bacterial cell contents occurs [39]. In this study, the possible mechanisms of action of the most potent EO, thyme EO, was investigated against acne-causing bacteria. It was concluded that thyme EO had a bactericidal and anti-biofilm activity against *C. acnes* and *S. epidermidis*. As demonstrated, thyme EO exerted its antimicrobial activity by affecting the cell membrane of acne-associated bacteria where a leakage of cytoplasmic components was observed. It was evidenced that thyme EO caused the loss of nucleic acids, increased potassium permeability and leakage of intracellular components (Figure 4 and Figure 5). The mechanism of action of the selected EO results was summarized in Figure 10. The SEM results confirmed the anti-biofilm effect of thyme EO on both *C. acnes* and *S. epidermidis* biofilms. In fact, Ref. [40] showed that thymol-rich oregano EO caused injury to *Escherichia coli* and *Bacillus Subtilis* by disrupting the cell membrane where a lack of cytoplasm was observed.

The in vitro studies corroborated that thyme EO was the most potent antimicrobial; therefore, it was prepared as a nanoemulsion formula to be assessed as an anti-acne agent in vivo. Nanoemulsions offer a solution for the low solubility of EO and enhance its application into the skin. In the current study, thyme EO at its double MIC (0.053 mg/mL) and 1% clindamycin (standard antibiotic) were formulated into nanoemulsions. The low-energy method was used to prepare the nanoemulsion [22] and the formulae were used to assess thyme EO in vivo using an acne animal ear mouse model.

To assess the efficacy of anti-acne agents, the suppression of inflammation and decease in bacterial load along with the analysis of histopathological tissues are evaluated in acne animal models [25,27,41,42]. The healing effect of thyme EO was demonstrated through the reduction in comedonal lesions, the reduction in ear thickness, the histological examination and the reduction in NF-KB and inflammatory mediators in thyme EO-treated mice ear tissue (Figure 9). Additionally, thyme EO nanoemulsion significantly lowered the *C. acnes* bacterial load. The in vivo mice model results confirmed the antimicrobial and anti-inflammatory activities of thyme EO against *C. acnes*. To the best of our knowledge, this is the first study to report thyme EO nanoemulsion as a possible alternative anti-acne therapeutic agent.

## 5. Conclusions

Microbiological screening showed that thyme, tea tree and clove EOs inhibited the growth of *C. acnes* and *S. epidermidis.* The strongest and fastest bactericidal effect was recorded with thyme EO, where a total elimination of the initial bacterial inoculum after 10 h and 6 h of exposure against *C. acnes* and *S. epidermidis*, respectively, was observed. In addition, thyme EO exhibited the greatest anti-biofilm activity. The antimicrobial activity of thyme EO was exerted by affecting the cell membrane of acne-associated bacteria causing the leakage of its cytoplasmic components. The TEM results showed morphological alterations of *C. acnes* and *S. epidermidis* post-treatment with the MBC of thyme EO. The GC-MS analysis of thyme EO proved that thymol was the main phenolic compound of the oil. In this study, thyme EO was found to be the most potent antimicrobial EO and was formulated as a nanoemulsion topical dosage form. When tested in an acne-induced BALB/c model, this nanoemulsion had healing effects through the reduction in comedonal lesions, the reduction in ear thickness, and the reduction in NF-KB and inflammatory mediators when compared to clindamycin. Additionally, the thyme nanoemulsion significantly lowered the *C. acnes* bacterial load. In conclusion, the in vivo mice model results confirmed the antimicrobial and anti-inflammatory activities of thyme EO against *C. acnes*.

## Figures and Tables

**Table 1:** List of the EOs, their common names, family names and part of the plant used.

Common Name	Latin Name	Family Name	Part of Plant Used
Tea tree	*Melaleuca alternifolia*	*Myrtaceae*	Buds
Clove	*Syzygium aromaticum*	*Myrtaceae*	Buds
Thyme	*Thymus vulgaris*	*Lamiaceae*	Leaves
Mentha	*Mentha spicate*	*Lamiaceae*	Leaves
Basil	*Ocimum basilicum*	*Lamiaceae*	Leaves and flowering tops

**Table 2:** The MIC of the tested EOs against *C. acnes* and *S. epidermidis* using the broth microdilution method.

Minimum Inhibitory Concentrations (MIC)		
EO	*C. acnes* (g/mL)	*S. epidermidis* (g/mL)
Tea tree	0.053	0.053
Clove	0.137	0.137
Thyme	0.026	0.053
Mentha	0.125	0.107
Basil	0.220	0.110

**Table 3:** The MBC of the tested EOs against *C. acnes* and *S. epidermidis* using the broth microdilution method.

Minimum Bactericidal Concentration (MBC)		
EO	*C. acnes* (g/mL)	*S. epidermidis* (g/mL)
Tea tree	0.053	0.053
Clove	0.137	0.137
Thyme	0.026	0.053
Mentha	0.215	0.107
Basil	0.220	0.110

**Table 4:** GC-MS analysis of volatile compounds in thyme EO.

Compound	Thyme EO Content (%)	Retention Time (Minutes)	Retention Index
*β*-Myrcene	0.38	9.026	872
*α*-Terpinolene	0.33	9.789	918
*o*-Cymene	4.17	10.096	929
Limonene	0.18	10.195	817
*γ*-Terpinene	1.21	11.122	941
*α*-Ocinene	0.60	12.405	897
Isoborneol	0.35	14.465	856
(+)-4-Carene	0.50	14.812	820
Carvacrol	2.18	18.708	926
Thymol	75.46	18.299	939
Caryophyllene	13.40	21.797	951
Humulene	0.28	22.689	902
Total identified compounds	99.04%		
Phenolic compounds	77.64%		
Terpenoid compounds	21.4%		

**Table 5:** The minimum biofilm inhibitory and eradication concentration of the screened EO against *S. epidermidis*.

EO	Minimum Biofilm Inhibitory Concentration (g/mL)	Minimum Biofilm Eradication Concentration (g/mL)
Thyme	0.026	0.053
Tea tree	0.107	No effect
Clove	0.274	0.274

**Table 6:** Comparison of signs of inflammation in the three mice groups.

Sign of Inflammation	Blank Formula-Treated Ear Tissue	1% Clindamycin-Treated Ear Tissue	Thyme EO-Treated Ear Tissue
Edema	++	+ ^1^	- ^2^
Vascular congestion	+	-	-
Inflammatory cells infiltration	++	Few	Markedly reduced

^1^ means mild increase, while two plus signs means marked increase, ^2^ means not found or detected.

## Data Availability

The raw data generated during the current study are available from the corresponding author upon request.

## Supplementary Materials

The following supporting information can be downloaded at: https://www.mdpi.com/article/10.3390/microorganisms10091874/s1, Figure S1: GC-MS chromatogram of tea tree EO; Figure S2: GC-MS chromatogram of clove EO; Figure S3: GC-MS chromatogram of thyme EO; Figure S4: Scanning electron microscopy (SEM) of thyme EO antibiofilm activity; Figure S5: Size distribution by intensity of the developed thyme oil nanoemulsion; Figure S6: TEM image of the developed thyme oil nanoemulsion, confirming that the formulation size is nanoparticle; Table S1: GC-MS analysis of volatile compounds in tea tree EO; Table S2: GC-MS analysis of volatile compounds in clove EO.
