# Supplementary material for: Antibacterial and Anti-Inflammatory Activities of Thymus vulgaris Essential Oil Nanoemulsion on Acne Vulgaris
Source: Microorganisms. 2022 Sep 19;10(9):1874. doi: 10.3390/microorganisms10091874 (PMC9503056; doi:10.3390/microorganisms10091874)
Supplement: Supplementary file 1 [file microorganisms-10-01874-s001.zip › microorganisms-1884084-supplementary.pdf]

Supplementary material

Table S1. GC-MS analysis of volatile compounds in tea tree EO

Compound	Tea tree EO Content (%)	Retention Time (minutes)	Retention Index
α-Pinene	0.43	7.294	918.1
Terpinolene	1.51	9.793	1068
o-Cymene	4.01	10.06	1085
Eucalyptol	3.25	10.25	1098
γ-Terpinen	16.70	11.16	1162
α-Terpinolene	1.41	12.04	1224.8
4-Terpinenyl acetate	66.82	14.93	1456
α-Terpineol	2.06	15.24	1481
Alloaromadendrene	0.68	22.3	1883
Aromadendrene	0.40	23.78	1960
Total identified compounds		97.27%	
Phenolic compounds		72.13%	
Terpenoid compounds		25.14%	

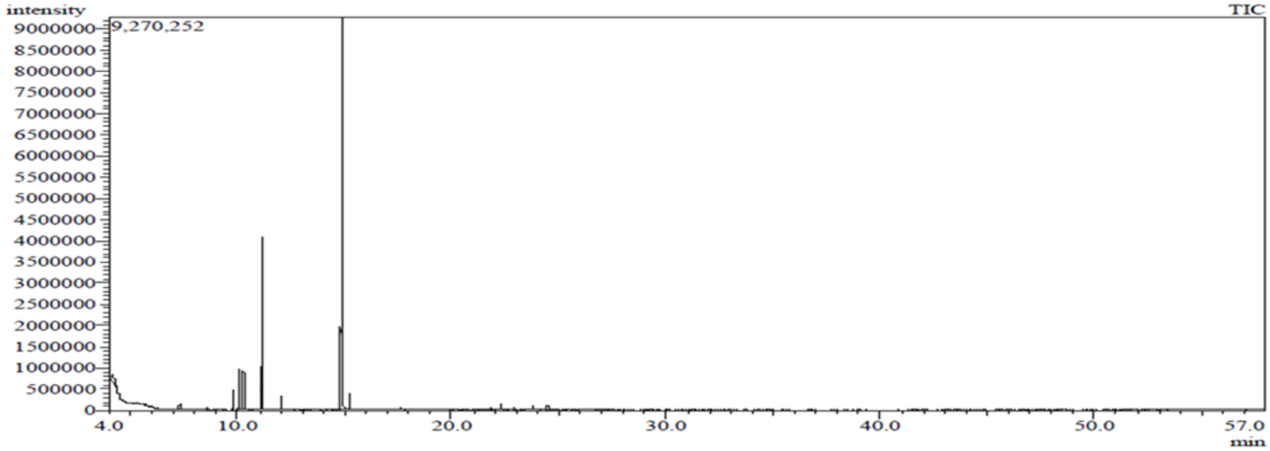

Figure S1. GC-MS Chromatogram of tea tree EO

Table S2. GC-MS analysis of volatile compounds in clove EO

Compound	Clove EO Content (%)	Retention Time (minutes)	Retention Index
Isoeugenol	1.70	20.175	1772
Eugenol	78.61	20.334	1781
Caryophyllene	11.94	21.799	1857
Humulene	1.71	22.694	1904
σ-Cardinene	0.56	24.473	1996.7
Eugenyl acetate	1.07	24.59	2003.4
Caryophyllene oxide	1.01	26.031	2037.9
Total identified compounds	96.6%		
Phenolic compounds	81.38%		
Terpenoid compounds	15.22%		

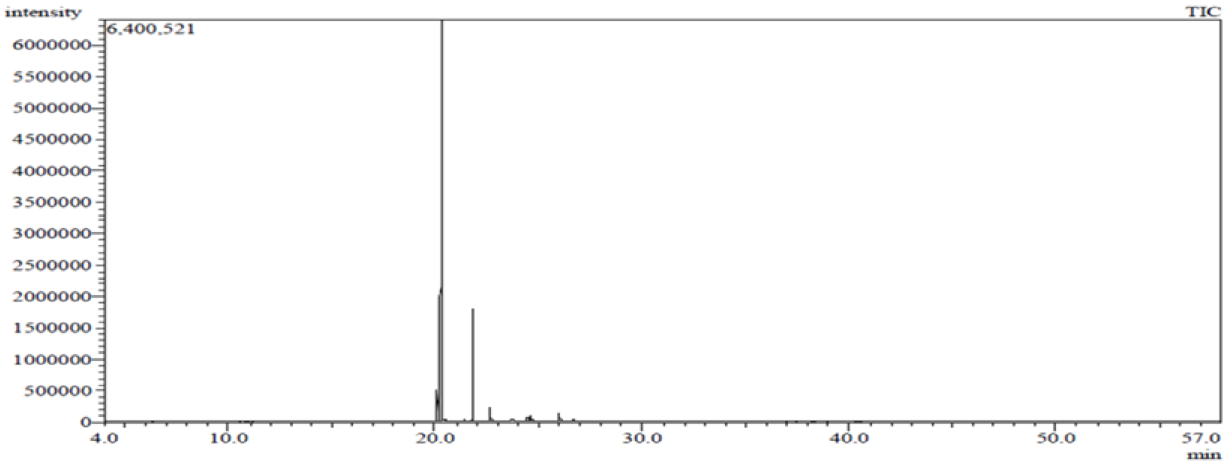

Figure S2. GC-MS Chromatogram of clove EO

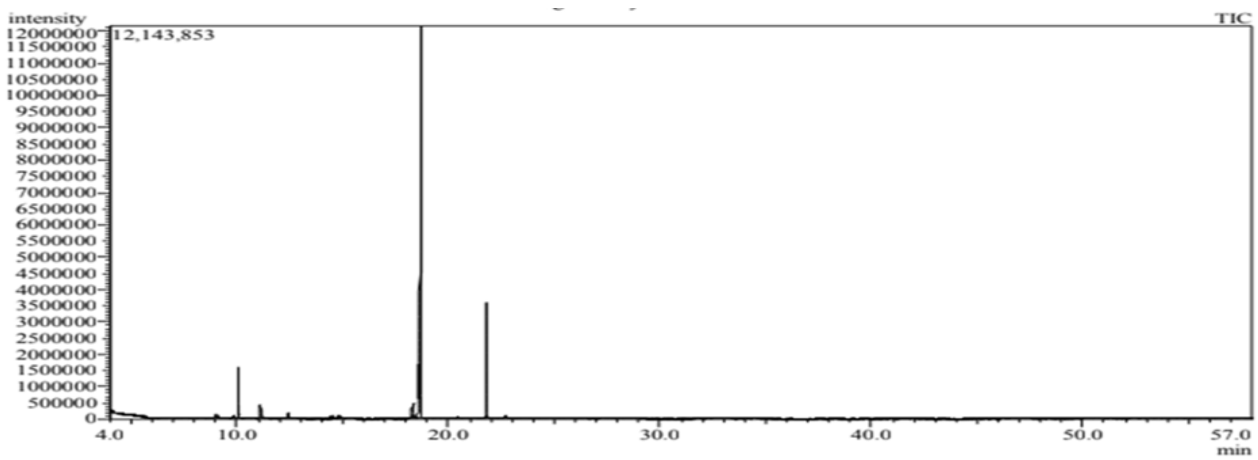

Figure S3. GC-MS Chromatogram of thyme EO

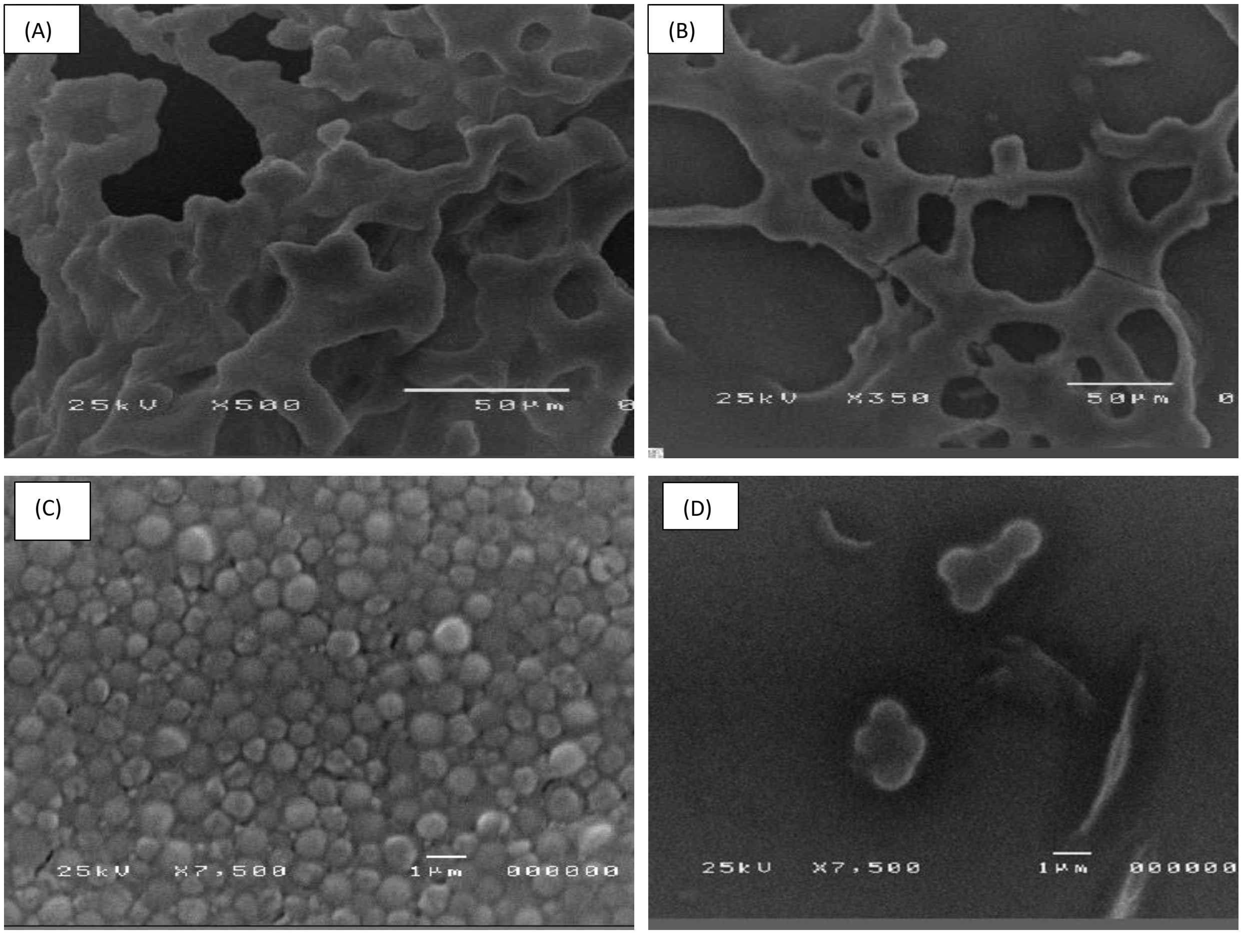

Figure S4. Scanning electron microscopy (SEM) of thyme EO antibiofilm activity showing A) C. acnes untreated biofilm, B) C. acnes biofilm treated with thyme, C) S. epidermidis untreated biofilm and D) S. epidermidis biofilm treated with thyme EO at a magnification of 5000x-7500x. Notice the reduction in cellular adhesion of both bacteria after treatment with thyme EO.

			Size (d.n...	% Intensity	Width (d.n...
Z-Average (d.nm): 77.32		Peak 1:	106.9	89.2	46.21
PdI: 0.386		Peak 2:	15.43	7.3	3.045
Intercept: 0.971		Peak 3:	4514	3.5	872.6
Result quality Good					

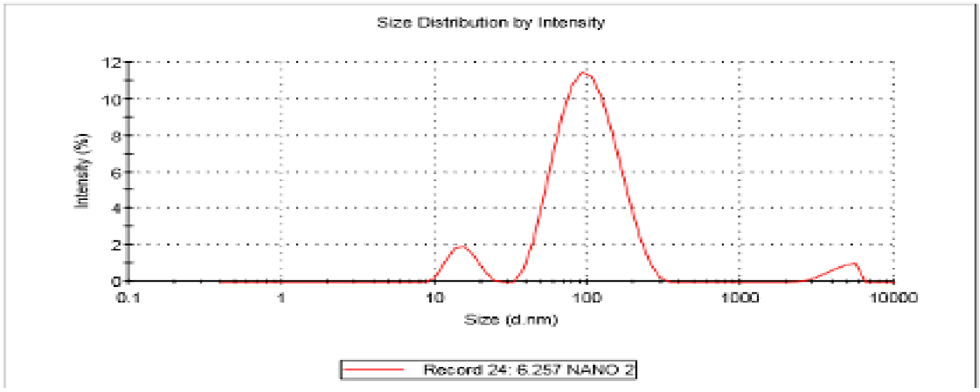

Figure S5. Size distribution by intensity of the developed thyme oil nanoemulsion

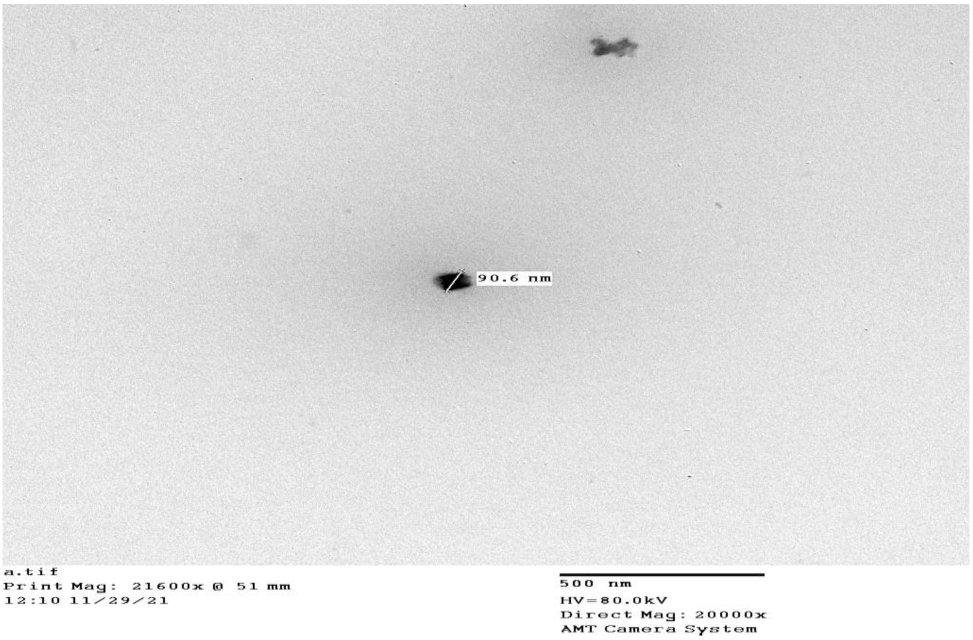

Figure S6. TEM image of the developed thyme oil nanoemulsion confirming that the formulation size is nano particle.
